# Use of Different Cardioplegia Solutions in Heart Valve Surgery

**DOI:** 10.21470/1678-9741-2023-0368

**Published:** 2024-05-13

**Authors:** Mesut Engin, Bişar Amaç, Mustafa Abanoz

**Affiliations:** 1 Department of Cardiovascular Surgery, University of Health Sciences, Bursa Yuksek Ihtisas Training and Research Hospital, Yıldırım, Bursa, Turkey. E-mail: mesut_kvc_cor@hotmail.com; 2 Department of Perfusion, University of Health Sciences, Mehmet Akif Inan Training and Research Hospital, Sanliurfa, Turkey; 3 Department of Cardiovascular Surgery, University of Health Sciences, Mehmet Akif Inan Training and Research Hospital, Sanliurfa, Turkey

Dear Editor,

We have read the article by Kantathut et al.^[1]^ entitled “Comparison of Single-Dose Cardioplegia in Valvular Heart Surgery: Lactated Ringer’s-Based del Nido *vs.* HistidineTryptophan-Ketoglutarate Cardioplegia Solution” with great interest. First of all, we congratulate the authors for their valuable contribution. However, we would like to discuss some points about cardioplegia solutions and the risks and benefits associated with them.

Del Nido cardioplegia solution (dNCS) was first used in congenital heart surgery and has also been used in adult heart surgery since 2003. The main liquid component in the original solution is Plasma-Lyte A and it can be modified by using other liquids (balanced electrolyte solution or lactated Ringer, as in the abovementioned study)^[1]^. Its effectiveness has been demonstrated in many studies in adult cardiac surgery^[2,3]^. However, the lack of a standard preparation, the high potassium content, and the risks of lidocaine toxicity in repeated doses are its disadvantages^[4]^. Custodiol® is used in complex cardiac surgery and transplant surgery in many centers with a single dose.

In that study, Kantathut et al. investigated the effectiveness of two different cardioplegia solutions in heart valve surgery in 71 adult patients. As a result, they concluded that the modified dNCS they prepared was as safe as Custodiol®^[1]^. However, we would like to draw attention to some perioperative variables of the study. Thus, we believe that it may be more descriptive for future studies. What does the “Chronic kidney disease” stated in the preoperative features mean? How many mg/dl above the creatinine value is used for this expression? All patients underwent valve surgery, and preoperative atrial fibrillation (AF) rates were given as 29.7% and 29.4%. In the postoperative period, this rate was found to be 7.6% and 20.8%. In our clinic, we routinely apply cryoablation or radiofrequency ablation methods to patients with preoperative AF. Were any ablation methods used in the patients with preoperative AF in the cited study? This may affect the comparison of the two groups in terms of arrhythmia.

Renal failure is an important problem in the postoperative period and can be detected at a rate of up to 40%^[5,6]^. Renal failure was detected in only one (0.014%) patient in the authors’ study group. To what do they attribute this situation? Or how did they define postoperative renal failure (creatinine > 4 mg/dl or hemodialysis)?

Finally, we think that the “Red cell transfusion, n, %” variable in the study may mislead us. Blood transfusion was required in 22 (64.71%) patients in the Custodiol® group and 14 (37.84%) patients in the dNCS group. The number of patients receiving blood transfusion was found to be significantly higher in the Custodiol® group (*P*=0.024). We believe that it would be beneficial to clearly state the patients’ preoperative hemoglobin values, autologous prime status in cardiopulmonary bypass systems, and blood transfusion indications when evaluating the necessity of blood transfusion^[7,8]^. It may also be better to use this variable as a numeric variable. For example, it was concluded that fewer patients in the dNCS group required blood transfusion, but a greater amount of red cell transfusion may have been performed.

## References

[1] Kantathut N, Luangpatom-Aram K, Khajarern S, Leelayana P, Cherntanomwong P. (2023). Comparison of single-dose cardioplegia in valvular heart surgery:
lactated ringer's-based del nido vs. histidine-tryptophan-ketoglutarate
cardioplegia solution. Braz J Cardiovasc Surg.

[2] Kağan As A, Engin M, Amaç B, Aydın U, Eriş C, Ata Y (2021). Effect of del nido cardioplegia use on kidney injury after
coronary bypass operations. Rev Assoc Med Bras (1992).

[3] Eris C, Engin M, Erdolu B, Kagan As A. (2022). Comparison of del nido cardioplegia vs. blood cardioplegia in
adult aortic surgery: is the single-dose cardioplegia technique really
advantageous?. Asian J Surg.

[4] Amaç B, Selçuk M, Bölükbaş S, Kahraman F, As AK, Savran M (2022). Use of del Nido cardioplegia in adult cardiac
surgery. Eur Res J.

[5] Engin M. (2020). Are pre and postoperative platelet to lymphocyte ratio and
neutrophil to lymphocyte ratio associated with early postoperative AKI
following CABG?. Braz J Cardiovasc Surg.

[6] Harky A, Joshi M, Gupta S, Teoh WY, Gatta F, Snosi M. (2020). Acute kidney injury associated with cardiac surgery: a
comprehensive literature review. Braz J Cardiovasc Surg.

[7] Faria LB, Mejia OV, Miana LA, Lisboa LAF, Manuel V, Jatene MB (2021). Anemia in cardiac surgery - can something bad get
worse?. Braz J Cardiovasc Surg.

[8] Paiva PP, Leite FM, Antunes PE, Antunes MJ. (2021). Risk-prediction model for transfusion of erythrocyte concentrate
during extracorporeal circulation in coronary surgery. Braz J Cardiovasc Surg.

